# Population-specific genetic differences of acute coronary syndrome in Han and Uyghur Chinese

**DOI:** 10.3389/fphar.2025.1588658

**Published:** 2025-08-15

**Authors:** Hongmei Lai, Jinhang Zhu, Jing Tao, Zitong Guo, Xiaolin Yu, Xin Shen, Ting Wang, Ying Wang, Huan Cai, Xiao Cai, Zhenbang Wei, Yining Yang

**Affiliations:** ^1^ The Cardiac and Panvascular Medicine Diagnosis and Treatment Center, People’s Hospital of Xinjiang Uyghur Autonomous Region, Xinjiang, China; ^2^ Xinjiang Key Laboratory of Cardiovascular Homeostasis and Regeneration Research, Xinjiang, China; ^3^ Department of Data and Analytics, WuXi Diagnostics Innovation Research Institute, Shanghai, China

**Keywords:** acute coronary syndrome, genetic association, clinical indicators, personalized medicine, predictive modeling

## Abstract

**Background:**

Acute coronary syndrome (ACS) is a critical cardiovascular condition with diverse clinical presentations, necessitating personalized therapeutic approaches. This study explores the genetic variation associated with ACS subtypes in the Han and Uyghur Chinese populations to support the development of precision medicine approaches tailored to ethnic-specific genetic backgrounds.

**Methods:**

A total of 985 ACS patients (668 Han and 317 Uyghur Chinese) representing different ACS subtypes were enrolled. Clinical characteristics and 66 genetic polymorphisms were analyzed. Statistical analyses were conducted to identify differences in genetic variants and clinical features across ACS subtypes and ethnic groups.

**Results:**

Significant clinical and genetic differences were observed between ACS subtypes and between ethnic groups. In the Han population, polymorphisms in *CYP2D6* and *PTGER3* were significantly associated with ACS subtypes (*p* ≤ 0.05). In the Uyghur population, six genes—*ACY3*, *CACNA1C*, *CYP2C9*, *CYP2C19*, *CYP4F2*, and *VKORC1*—showed significant associations (*p* ≤ 0.05). These findings indicate distinct genetic landscapes across the two ethnic groups. Furthermore, population-specific associations between genetic variants and artery narrowing were identified. Predictive models integrating clinical and genetic features achieved an area under the curve (AUC) of 0.832 [95% confidence intervals (CI): 0.774–0.889] in Uyghur patients and 0.674 (95% CI: 0.626–0.722) in Han patients, indicating a higher internal AUC of these genetic markers in the Uyghur population.

**Conclusion:**

This study highlights ethnic differences in the genetic architecture of ACS. The result also underscores the need for population-specific strategies in risk stratification and treatment. The identified genetic markers and predictive models may guide future research on ethnicity-specific risk stratification.

## Introduction

Acute coronary syndrome (ACS) is a critical medical condition characterized by a sudden blockage of blood flow to the heart, typically caused by the rupture of an atherosclerotic plaque in a coronary artery. ACS encompasses a spectrum of clinical presentations, including unstable angina, non-ST-segment elevation myocardial infarction, and ST-segment elevation myocardial infarction (Koufaki et al., 2023; Sarhan et al., 2023). This syndrome poses a significant threat to cardiovascular health and demands prompt medical attention to prevent severe complications, such as myocardial infarction and cardiac death. In recent years, the field of pharmacogenomics has gained prominence in the study of ACS. Genetic research on the drug response explores the influence of genetic variations on the individual response to drugs. This approach holds great promise in optimizing therapeutic strategies for ACS patients as genetic factors can significantly impact drug metabolism, efficacy, and adverse reactions (Angulo-Aguado et al., 2021; Ellithi et al., 2020). Genetic studies in ACS have shown that polymorphisms may affect the pharmacokinetics and pharmacodynamics of medications commonly used in the management of ACS, such as antiplatelet agents, statins, and beta-blockers. By elucidating the interplay between genetic factors and drug response, researchers aim to identify biomarkers that can aid in predicting individual patient responses to specific medications, allowing for more personalized and effective treatment approaches (Tardif et al., 2022; He et al., 2021).

Understanding the population-specific genetic variation related to ACS in different populations is crucial for developing personalized therapeutic strategies (Yang et al., 2022; Kuo et al., 2022). The Han Chinese population represents a significant demographic due to its large size and genetic diversity (Qi et al., 2020). The genetic polymorphisms within this population have been implicated in various cardiovascular conditions, making it a pertinent focus for genetic studies related to ACS (Zhou et al., 2022). Several candidate genes associated with ACS have been identified, and understanding their polymorphic variations among the Han Chinese populations is essential for predicting susceptibility to ACS, optimizing treatment outcomes, and minimizing adverse drug reactions. More research is needed to bridge the knowledge gap in genetic research related to ACS within these populations, contributing valuable insights to the development of precision medicine approaches tailored to the genetic profiles of Han Chinese individuals (Zheng et al., 2020; Zhang et al., 2020).

The Uyghur population has a history of extensive interaction with both Eastern Asian and European populations. As a result, Uyghurs exhibit an admixture of Eastern and Western anthropological and genetic traits (Bian et al., 2016). ACS has been observed to carry a high mortality rate within the Uyghur Chinese population, which is further characterized by its unique genetic traits, cultural practices, and environmental exposures. Therefore, Uyghur individuals represent an important yet understudied demographic in the context of genetic research (Li et al., 2021; Wang et al., 2021; Zhao et al., 2023). The intricate relationship between genetic polymorphisms and ACS susceptibility has been widely acknowledged, and understanding the genetic profile of ACS within the Uyghur Chinese population is pivotal for advancing personalized medicine strategies (Li et al., 2021). Limited studies have focused specifically on the genetic variations in candidate genes associated with ACS in the Uyghur population, necessitating a dedicated effort to bridge this knowledge gap. More studies are needed to contribute valuable insights that can inform more tailored and effective therapeutic approaches for individuals of Uyghur descent facing ACS risks (Yu et al., 2021).

In the present study, we hypothesize that genetic biomarker profiles differ significantly between Han and Uyghur Chinese populations and across subtypes of ACS. To test this, we aim to identify population-specific genetic polymorphisms associated with ACS (Koufaki et al., 2023), compare the distribution of genetic markers across ACS subtypes within each ethnic group (Sarhan et al., 2023), and evaluate the predictive value of these biomarkers in distinguishing ACS subtypes (Angulo-Aguado et al., 2021). Our goal is to provide insights that support the development of personalized treatment strategies tailored to the genetic backgrounds of diverse Chinese populations.

## Materials and methods

### Study participants

This is a single-center study. We enrolled patients aged 18 years or older with angiographically confirmed coronary artery disease at People’s Hospital of Xinjiang Uyghur Autonomous Region (Xinjiang, China), between January 2020 and December 2022. Patients who were hospitalized for ACS were included in our study. ACS was defined as ST elevation myocardial infarction (STEMI), non-ST elevation myocardial infarction (NSTEMI), or unstable angina pectoris (UA), according to the International Statistical Classification of Diseases and Related Health Problems, 10th Revision, code of I20 (angina pectoris) or I21 (acute myocardial infarction). Finally, 985 patients were collected, including 131 cases of STEMI, 119 cases of NSTEMI, and 735 cases of UA.

### Selection of candidate SNPs

A total of 66 single-nucleotide polymorphisms (SNPs) across 32 genes were selected to form a cardiovascular gene panel. The selection process was based on multiple criteria, including recommendations from clinical guidelines, expert consensus, the PharmGKB database developed by the U.S. National Institutes of Health, and relevant peer-reviewed literature. We prioritized SNPs based on their documented associations with drug response or metabolism, especially for drugs commonly used in the treatment for cardiovascular and cerebrovascular diseases. These included antiplatelet agents, anticoagulants, lipid-lowering drugs, and antihypertensive medications. Additional considerations included allele frequency distributions in the Chinese population and the level of evidence supporting each drug–gene interaction. Accordingly, the panel comprises variants that have shown pharmacologic or disease associations in prior literature or curated databases; some have direct functional evidence, whereas others serve as tag SNPs that track causal alleles in high linkage disequilibrium. This selection process ensured clinical relevance and population-specific applicability of the 66 SNPs analyzed in this study. The detailed information of these 66 polymorphisms is shown in Supplementary Table 1.

### Genomic DNA extraction and SNP detection

We collected 2 mL of peripheral venous blood using an EDTA anticoagulation tube, extracted genomic DNA using the KingFisher Flex Nucleic Acid Extractor (Thermo Fisher, Waltham, United States), and detected DNA concentration and purity using NanoDrop 2000 (Thermo Fisher, Waltham, United States). DNA concentration was maintained at ≥ 10 ng/μL, with an A260/A280 ratio between 1.7 and 2.0.

### SNP genotyping

SNPs were divided into three wells for multiple PCRs, and the target fragment containing the SNP to be detected was amplified. The reaction system of multiplex PCR (5 μL) consisted of the following: 0.5 μL 10x PCR buffer (Agena Bioscience, San Diego, United States), 0.4 μL MgCl_2_ (Agena Bioscience, San Diego, United States), 0.1 μL dNTP mix (Agena Bioscience, San Diego, United States), 0.2 μL PCR enzyme (Agena Bioscience, San Diego, United States), 1.0 μL Primer mix (500 nM each) (Invitrogen, Carlsbad, United States), 0.8 μL nuclease-free water (Invitrogen, Carlsbad, United States), and 2 μL genomic DNA. PCR was conducted using the Applied Biosystems Veriti 96-Well (Thermo Fisher, Waltham, United States) under the following standard conditions: denaturation at 95°C for 2 min, followed by 45 cycles at 95°C for 30 s, annealing temperature 60°C for 60 s, extending 72°C for 60 s, and a final extension at 72°C for 5 min. Then, we added 2 μL of the SAP reaction mixture after PCR amplification for dephosphorylating and degrading the remaining dNTP. The components of the SAP reaction mixture (2 μL) are as follows: 0.17 μL SAP buffer (Agena Bioscience, San Diego, United States), 0.30 μL SAP enzyme (Agena Bioscience, San Diego, United States), and 1.53 μL nuclease-free water (Invitrogen, Carlsbad, United States).The SAP reaction was conducted using the Applied Biosystems Veriti 96-Well (Thermo Fisher, Waltham, United States) under the following standard conditions: 37°C for 40 min, followed by one cycle at 85°C for 5 min. After the SAP reaction, we added 2 μL of the extension reaction mixture for the single-base extension reaction. Extension primers annealed to the 5′ flanking region of each SNP and incorporated a single base during extension. The components of the extension reaction mixture (2 μL) are as follows: 0.2 μL iPlex buffer plus (Agena Bioscience, San Diego, United States), 0.2 μL iPlex termination mix (Agena Bioscience, San Diego, United States), 0.04 μL iPlex pro enzyme (Agena Bioscience, San Diego, United States), 0.94 μL extend primers mix (Agena Bioscience, San Diego, United States), and 0.62 μL nuclease-free water (Invitrogen, Carlsbad, United States). The extension reaction was also conducted using the Applied Biosystems Veriti 96-Well (Thermo Fisher, Waltham, United States) under the following standard conditions: 95°C for 30 s, followed by 40 cycles of extension (95°C for 5 s, followed by five cycles at 52°C for 5 s and 80°C for 5 s) and 72°C for 3 min. The extended product was detected using a MassARRAY nucleic acid mass spectrometry system (Agena Bioscience, San Diego, United States), and the results were analyzed using MassARRAY Typer 4.1 software (Agena Bioscience, San Diego, United States).

### Power analysis

To evaluate whether the sample size was adequate for detecting differences in genotype distributions across ACS subtypes, we performed a *post hoc* power analysis using the pwr.chisq.test() function from the R package pwr (version 1.3.0). Based on an assumed medium effect size (Cohen’s w = 0.3), a significance level of 0.05, and 4 degrees of freedom (3 genotypes × 3 ACS subtypes), the analysis demonstrated sufficient power for both populations studied. The statistical power was >0.99 for the Han population (N = 668) and 0.995 for the Uyghur population (N = 317). The results indicated that the sample sizes were sufficient to detect meaningful differences in genotype distributions among ACS subtypes.

### Statistical analysis

The demographic and clinical characteristics of the patients were summarized using conventional descriptive statistics: n (%) for categorical variables and medians and quartiles (25% quantile and 75% quantile) for continuous variables. Categorical variables were analyzed using the chi-squared test and Fisher’s exact test, and multi-group comparisons of continuous variables were assessed using the Kruskal–Wallis test. A statistically significant difference was defined as a *p*-value < 0.05. To account for multiple comparisons in genetic association analyses, false discovery rate (FDR) correction using the Benjamini–Hochberg method was applied to the *p*-values. Decision tree models were constructed using recursive partitioning. Receiver operating characteristic (ROC) curves were constructed, and the area under the curve (AUC) and 95% confidence intervals (CIs) are reported as a measure of model performance. The sensitivity, specificity, positive predictive value (PPV), negative predictive value (NPV), and accuracy were used to show diagnostic accuracy. All data analyses were performed using R software 4.0.3 (R Foundation, Vienna, Austria).

## Results

### Clinical characteristics of participants in the Han and Uyghur Chinese populations

The study included 985 samples: 668 Han Chinese and 317 Uyghur Chinese individuals. There were 485 cases of unstable angina, 85 cases of non-ST-segment elevation myocardial infarction, and 98 cases of ST-segment elevation myocardial infarction in the 668 Han Chinese samples. There were 250 cases of unstable angina, 34 cases of non-ST-segment elevation myocardial infarction, and 33 cases of ST-segment elevation myocardial infarction in the 317 Uyghur Chinese samples. Age, sex, height, and weight did not show significant differences between the Han and the Uyghur population. Some important clinical indicators such as heart function classification, Killip classification, number of narrowed vessels, left main artery narrowing, left anterior descending narrowing, circumflex artery narrowing, right coronary narrowing, B-type natriuretic peptide (BNP), and ejection fraction (EF) were assessed. The statistical results showed significant differences in left anterior descending narrowing, BNP, and EF among ACS subtypes in the Han population (Table 1) and in cardiac function grade, BNP, and EF in the Uyghur population (Table 2).

**TABLE 1:** Demographics based on the Han Chinese population.

ACS type	NSTEMI, N = 85^a^	STEMI, N = 98^a^	UA, N = 485^a^	*p*-value^b^
Ethnicity				
Han	85 (100%)	98 (100%)	485 (100%)	
Age	64 (54, 73)	58 (48, 69)	61 (54, 69)	0.029
Gender				0.3
Man	68 (80%)	77 (79%)	355 (74%)	
Woman	17 (20%)	20 (21%)	126 (26%)	
Height	170 (165, 173)	170 (165, 174)	170 (162, 174)	0.7
Weight	73 (63, 80)	73 (64, 80)	74 (65, 81)	0.6
Cardiac function grade				0.079
1	3 (18%)	5 (26%)	10 (13%)	
2	3 (18%)	8 (42%)	33 (43%)	
3	7 (41%)	6 (32%)	29 (38%)	
4	4 (24%)	0 (0%)	4 (5.3%)	
Killip classification				0.090
1	21 (52%)	33 (51%)	5 (56%)	
2	11 (28%)	16 (25%)	1 (11%)	
3	6 (15%)	4 (6.2%)	3 (33%)	
4	2 (5.0%)	12 (18%)	0 (0%)	
Number of narrowed vessels	2.0 (1.5, 3.0)	2.0 (1.0, 3.0)	2.0 (1.0, 3.0)	0.12
Left main artery narrowing	10 (18%)	15 (27%)	70 (24%)	0.5
Left anterior descending narrowing	67 (100%)	80 (95%)	333 (88%)	0.002
Circumflex artery narrowing	50 (81%)	47 (71%)	248 (71%)	0.3
Right coronary narrowing	48 (77%)	53 (75%)	256 (73%)	0.8
BNP	183.7 (39.8, 458.0)	160.5 (66.5, 409.3)	40.1 (18.4, 106.3)	<0.001
EF	55.0 (50.0, 60.0)	50.0 (50.0, 56.0)	60.0 (57.0, 62.0)	<0.001

^a^ n (%); Median (IQR).

^b^ Kruskal–Wallis rank sum test; Pearson’s chi-squared test; Fisher’s exact test.

ACS, acute coronary syndrome; UA, unstable angina; NSTEMI, non-ST-segment elevation myocardial infarction; STEMI, ST-segment elevation myocardial infarction; BNP, B-type natriuretic peptide; EF, ejection fraction.

**TABLE 2:** Demographics based on the Uyghur Chinese population.

ACS type	NSTEMI, N = 34^a^	STEMI, N = 33^a^	UA, N = 250^a^	*p*-value^b^
Ethnicity				
Uyghur	34 (100%)	33 (100%)	250 (100%)	
Age	59 (55, 67)	55 (50, 62)	60 (54, 66)	0.069
Gender				0.3
Man	27 (79%)	30 (91%)	196 (79%)	
Woman	7 (21%)	3 (9.1%)	53 (21%)	
Height	166 (160, 174)	170 (167, 172)	169 (162, 172)	0.5
Weight	75 (70, 81)	78 (70, 86)	80 (72, 86)	0.3
Cardiac Function Grade				0.019
1	1 (12%)	1 (14%)	3 (6.5%)	
2	0 (0%)	2 (29%)	26 (57%)	
3	5 (62%)	3 (43%)	14 (30%)	
4	2 (25%)	1 (14%)	3 (6.5%)	
Killip Classification				0.3
1	8 (57%)	12 (50%)	4 (67%)	
2	4 (29%)	2 (8.3%)	1 (17%)	
3	0 (0%)	5 (21%)	1 (17%)	
4	2 (14%)	5 (21%)	0 (0%)	
Number of narrowed vessels	2.0 (2.0, 3.0)	1.0 (1.0, 2.0)	2.0 (1.0, 3.0)	0.2
Left main artery narrowing	4 (19%)	5 (25%)	20 (14%)	0.3
Left anterior descending narrowing	22 (81%)	26 (96%)	173 (86%)	0.2
Circumflex artery narrowing	22 (81%)	14 (64%)	136 (76%)	
Right coronary narrowing	18 (82%)	17 (71%)	132 (73%)	0.6
BNP	106.9 (49.6, 788.4)	121.0 (60.4, 424.8)	47.8 (24.6, 118.1)	<0.001
EF	58.0 (52.5, 60.0)	53.0 (50.0, 55.0)	60.0 (55.0, 61.2)	0.001

^a^ n (%); Median (IQR).

^b^ Kruskal–Wallis rank sum test; Pearson’s chi-squared test; Fisher’s exact test.

ACS, acute coronary syndrome; UA, unstable angina; NSTEMI, non-ST-segment elevation myocardial infarction; STEMI, ST-segment elevation myocardial infarction; BNP, B-type natriuretic peptide; EF, ejection fraction.

### Significant genetic loci among ACS subtypes in Han and Uyghur populations

**FIGURE 1:**
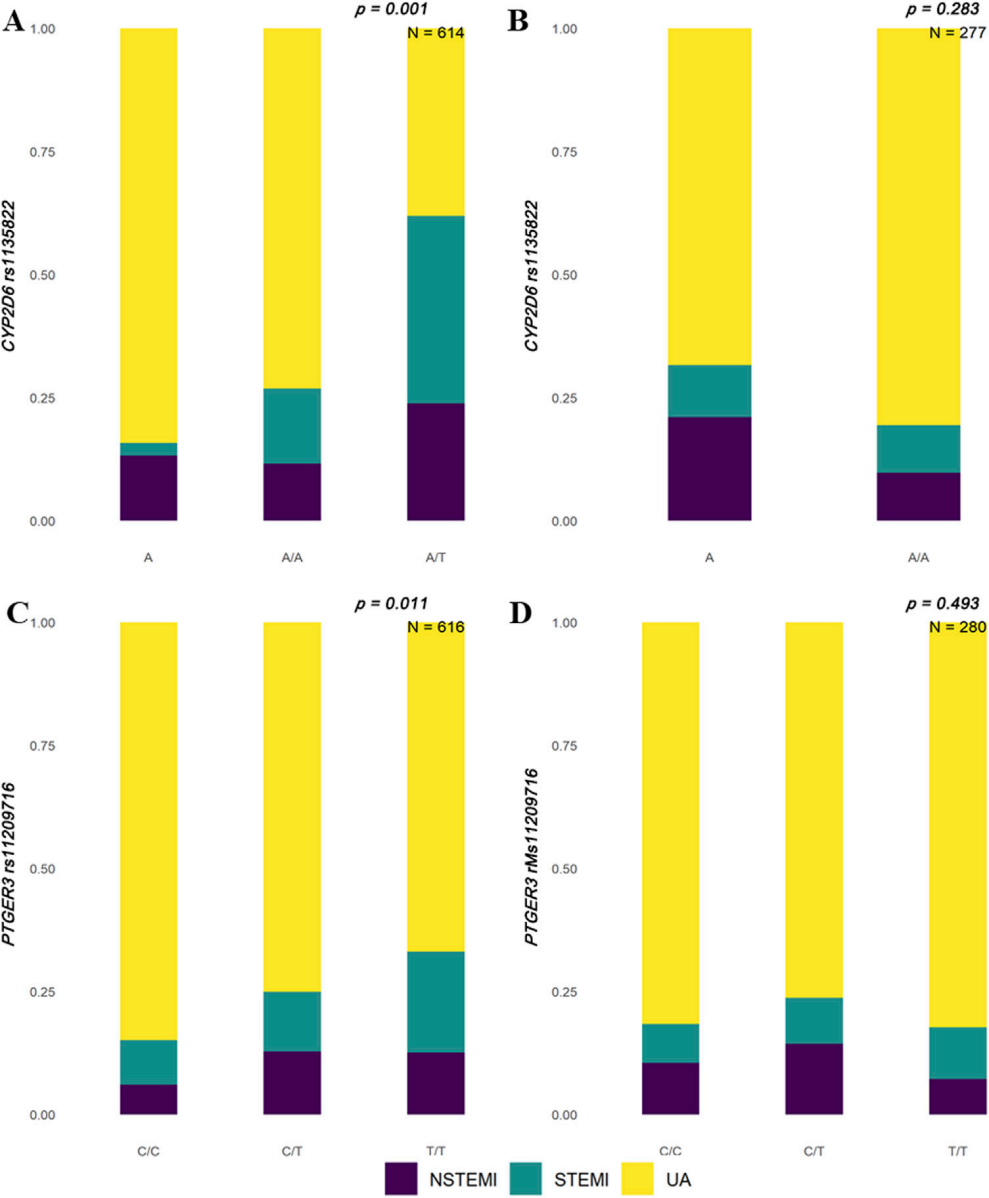
Distribution of ACS subtypes by *CYP2D6* and *PTGER3* genotypes in Han and Uyghur populations. Stacked bar plots illustrate the proportions of acute coronary syndrome (ACS) subtypes—non-ST-segment elevation myocardial infarction (NSTEMI, purple), ST-segment elevation myocardial infarction (STEMI, teal), and unstable angina (UA, yellow)—across different genotypes. **(A)** In the Han population, *CYP2D6* rs1135822 shows a significant association with ACS subtypes (*p* = 0.001, N = 614), with the A/T genotype displaying a higher proportion of STEMI cases. **(B)** In the Uyghur population, no significant association is observed for *CYP2D6* rs1135822 (*p* = 0.283, N = 277). **(C)** In the Han population, *PTGER3* rs11209716 also shows a significant association (*p* = 0.011, N = 616), with the T/T genotype enriched in UA cases. **(D)** In the Uyghur population, *PTGER3* rs11209716 does not reach statistical significance (*p* = 0.493, N = 280).

**FIGURE 2:**
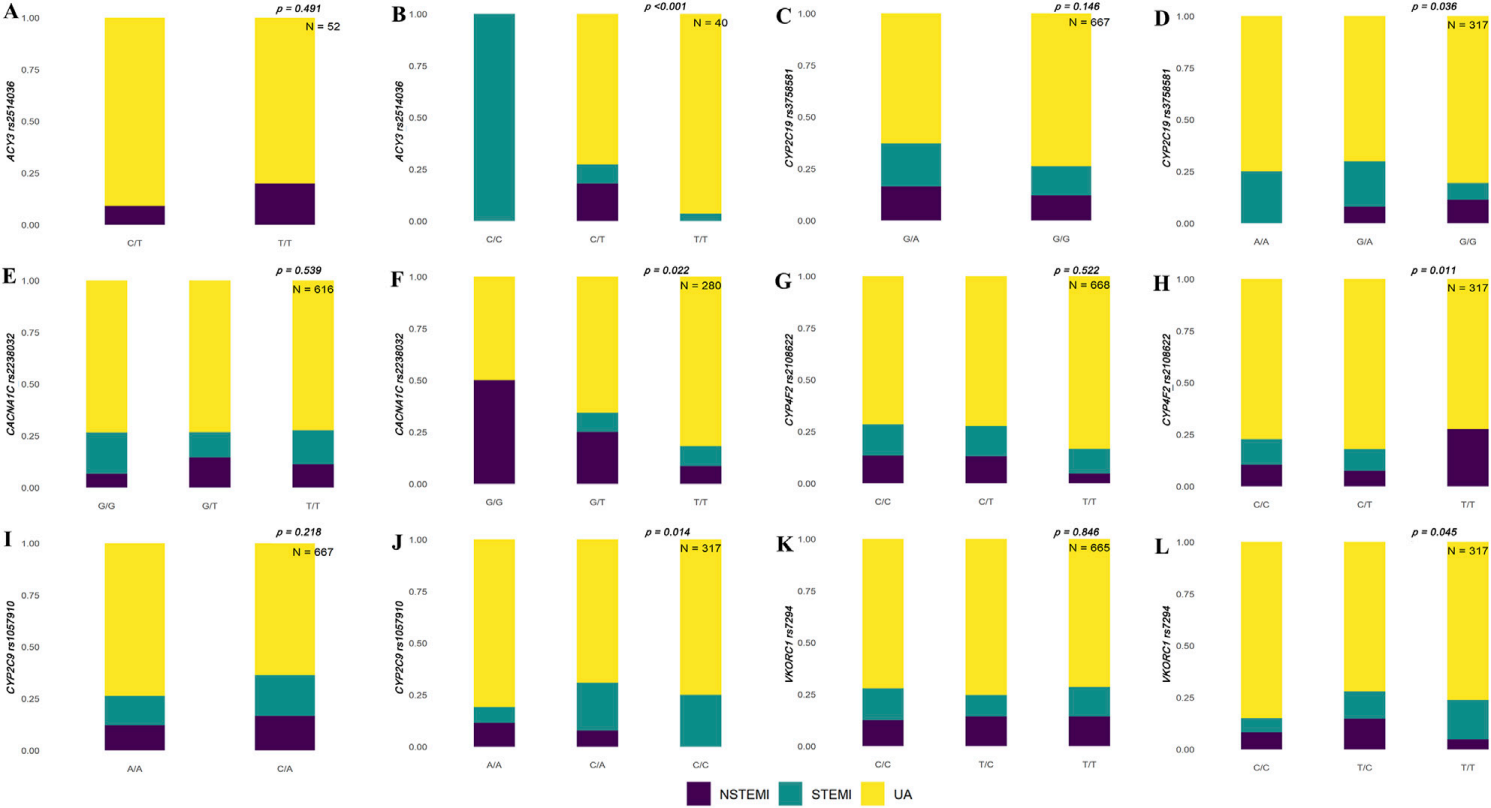
Population-specific associations between genetic variants and ACS subtypes in Han and Uyghur populations. Stacked bar plots show the distribution of non-ST-segment elevation myocardial infarction (NSTEMI, purple), ST-segment elevation myocardial infarction (STEMI, teal), and unstable angina (UA, yellow) across different genotypes for selected genetic loci. Panels on the left **(A, C, E, G, I, K)** represent results for the Han population, and panels on the right **(B, D, F, H, J, L)** represent the results for the Uyghur population. **(A, B)** *ACY3* rs2514036 shows a significant association with ACS subtypes in Uyghur individuals (*p* < 0.001, N = 40), but not in Han population (*p* = 0.491, N = 52). **(C, D)** *CYP2C19* rs3758581 is significantly associated in Uyghur (*p* = 0.036, N = 317), but not in Han population (*p* = 0.146, N = 667). **(E, F)** *CACNA1C* rs2238032 shows a significant association in Uyghur (*p* = 0.022, N = 280), but not in Han population (*p* = 0.539, N = 616). **(G, H)** *CYP4F2* rs2108622 is significant in Uyghur (*p* = 0.011, N = 317), but not in Han population (*p* = 0.522, N = 668). **(I, J)** *CYP2C9* rs1057910 shows significance in Uyghur (*p* = 0.014, N = 317), but not in Han population (*p* = 0.218, N = 667). **(K, L)** *VKORC1* rs7294 shows marginal significance in Uyghur (*p* = 0.045, N = 317), but not in Han population (*p* = 0.846, N = 665).

We identified significant differences in polymorphic loci of candidate genes in the Han and Uyghur populations. The results showed that rs1135822 in *CYP2D6* and rs11209716 in *PTGER3* were significantly different among ACS subtypes in the Han Chinese population, but not in the Uyghur Chinese population (Figure 1). Meanwhile, rs2514036 in *ACY3*, rs3758581 in *CYP2C19*, rs2238032 in *CACNA1C*, rs2108622 in *CYP4F2*, rs1057910 in *CYP2C9*, and rs7294 in *VKORC1* were significantly different among ACS subtypes in the Uyghur Chinese population, but not in the Han Chinese population (Figure 2). The complete chi-squared test results for all 66 candidate gene loci, including raw and FDR-adjusted *p*-values, are provided in Supplementary Table 2.

### Identification of significant clinical indicators associated with SNPs in Han and Uyghur ACS patients

**FIGURE 3:**
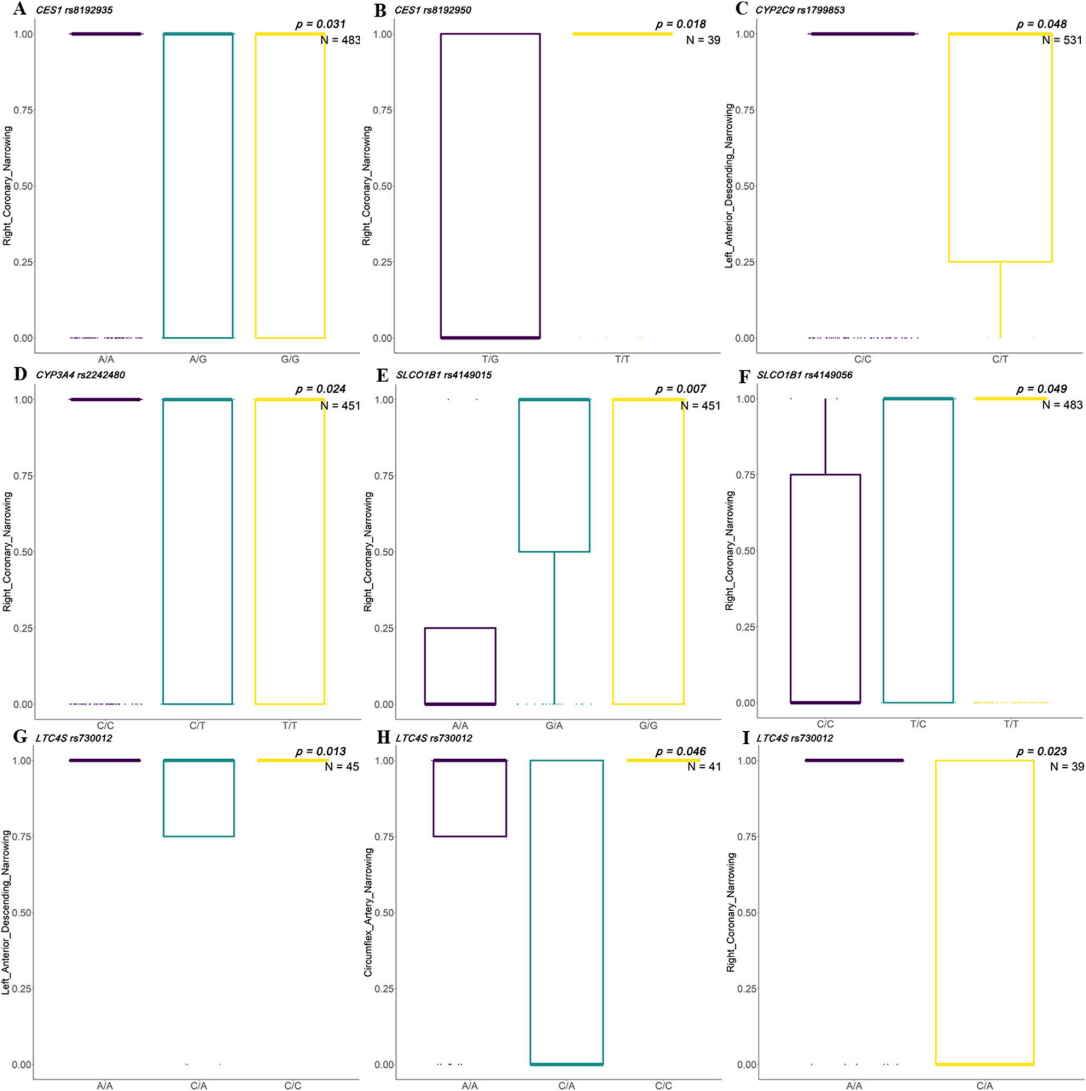
Associations between clinical indicators and SNP genotypes in Han Chinese patients with ACS. Box plots illustrate significant differences in artery narrowing percentages across genotypes for selected single-nucleotide polymorphisms (SNPs) among patients with acute coronary syndrome (ACS) in the Han population. Genotype groups are compared for their association with stenosis in three major coronary arteries: right coronary artery (RCA), left anterior descending artery (LAD), and left circumflex artery (LCX). **(A, B)** *CES1* variants rs8192935 and rs8192950 show significant genotype-based differences in RCA narrowing (*p* = 0.031, N = 483). **(C)** *CYP2C9* rs1799853 shows a significant association with LAD narrowing (*p* = 0.018, N = 39). **(D)** *CYP3A* rs2242480 shows a differential association with RCA narrowing (*p* = 0.048, N = 531). **(E, F)** *SLCO1B1* rs4149015 (*p* = 0.007, N = 451) and rs4149056 (*p* = 0.049, N = 483) exhibit a significant variation in RCA narrowing by genotype. **(G–I)** *LTC4S* rs730012 is associated with LAD (*p* = 0.013, N = 45), LCX (*p* = 0.046, N = 41), and RCA (*p* = 0.023, N = 39) narrowing, showing a genotype-dependent vascular impact across all three arteries.

Coronary artery narrowing is a major contributor to ACS development. This study examined the association between specific genetic variants and the degree of artery stenosis, identifying several polymorphisms that showed statistically significant differences in coronary artery narrowing (e.g., left anterior descending and right coronary) among different genotypes. In the Han Chinese population, rs8192935 and rs8192950 in *CES1* showed significant differences in right coronary artery narrowing. Different genotypes of rs1799853 in *CYP2C9* showed significant differences in left anterior descending narrowing. Different genotypes in rs2242480 in *CYP3A4* showed a significant association with right coronary narrowing. Different genotypes of rs4149015 and rs4149056 in *SLCO1B1* showed significant association with right coronary narrowing. Genotypes of rs730012 in *LTC4S* showed significant differences in narrowing of the left anterior descending, circumflex, and right coronary arteries (Figure 3).

**FIGURE 4:**
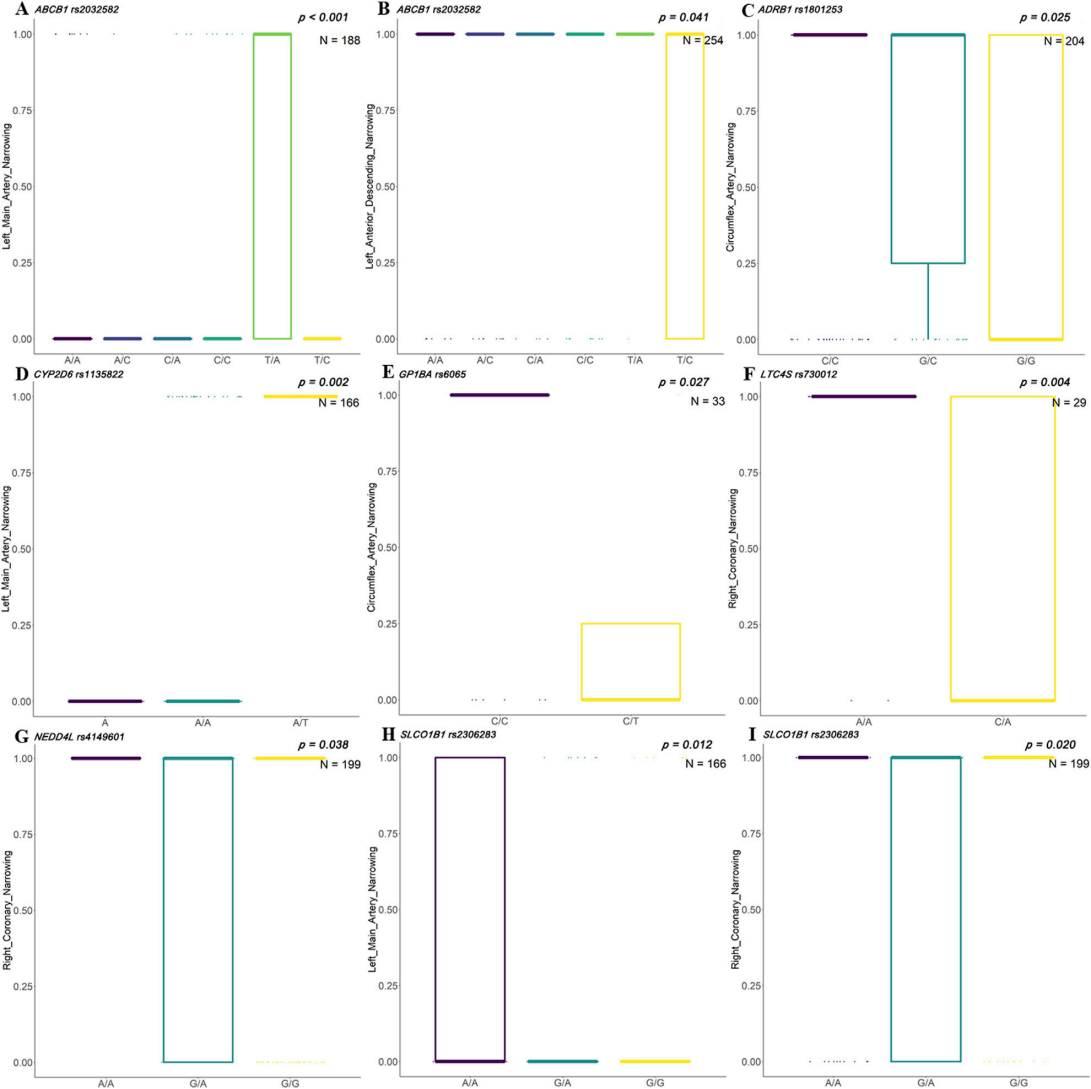
Associations between clinical indicators and SNP genotypes in Uyghur Chinese patients with ACS. Box plots illustrate the distribution of stenosis levels in different coronary arteries across genotypes of genetic single-nucleotide polymorphisms (SNPs) in acute coronary syndrome (ACS) patients of Uyghur descent. **(A, B)** *ABCB1* rs2032582 shows significant associations with left main artery narrowing (*p* < 0.001, N = 188) and left anterior descending (LAD) narrowing (*p* = 0.041, N = 254). **(C)** *ADRB1* rs1801253 is significantly associated with circumflex artery narrowing (*p* = 0.025, N = 204). **(D)** *CYP2D6* rs1135822 shows a strong association with left main artery narrowing (*p* = 0.002, N = 166). **(E)** *GP1BA* rs6065 demonstrates significance for circumflex artery narrowing (*p* = 0.027, N = 33). **(F)** *LTC4S* rs730012 shows significant genotype-specific variation in right coronary artery narrowing (*p* = 0.004, N = 29). **(G)** *NEDD4L* rs4149601 is associated with right coronary artery narrowing (*p* = 0.038, N = 199). **(H, I)** *SLCO1B1* rs2306283 is significantly associated with narrowing in both the left main artery (*p* = 0.012, N = 166) and right coronary artery (*p* = 0.020, N = 199).

In the Uyghur population, rs2032582 in *ABCB1* showed significant differences in narrowing of left main artery and left anterior descending artery. Genotypes of rs1801253 in *ADRB1* showed a significant association with circumflex artery narrowing. Genotypes of rs1135822 in *CYP2D6* showed a significant association with left main artery narrowing. Genotypes of rs6065 in *GP1BA* showed a significant association with circumflex artery narrowing. Genotypes of rs730012 in *LTC4S* and rs4149601 in *NEDD4L* showed a significant association with right coronary narrowing. Genotypes of rs2306283 in *SLCO1B1* showed a significant association with narrowing of the left main coronary artery and right coronary artery (Figure 4).

### Prediction of ACS subtypes using the polymorphism site and clinical indicators in Han and Uyghur Chinese populations

**FIGURE 5:**
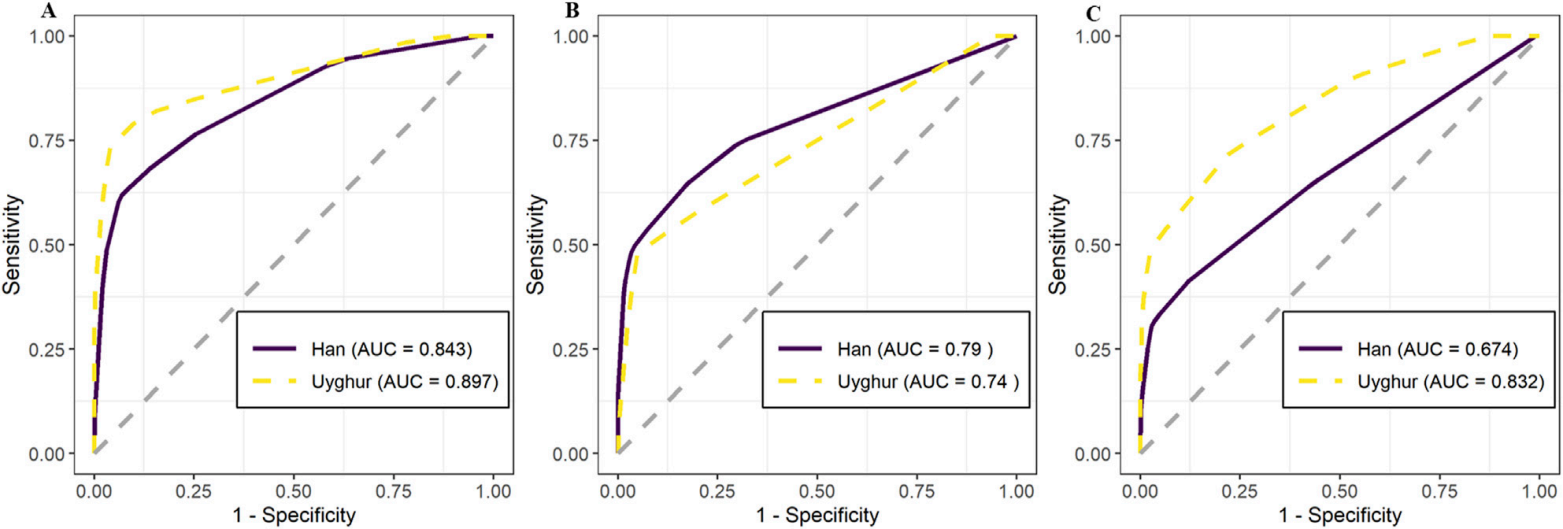
Predictive performance of models using clinical and genetic data to distinguish unstable angina (UA) from myocardial infarction (MI) in Han and Uyghur Chinese populations. Receiver operating characteristic (ROC) curves show the predictive accuracy of models across three input feature sets, with comparisons between Han (solid purple line) and Uyghur (dashed yellow line) populations. Model performance is quantified using area under the curve (AUC). **(A)** Combined model using both clinical indicators and genetic variants achieved high AUC values in both Han (AUC = 0.843) and Uyghur (AUC = 0.897) populations. **(B)** Model using only clinical indicators showed moderate performance: Han (AUC = 0.790) and Uyghur (AUC = 0.740). **(C)** Model using only genetic variants demonstrated a stronger predictive value in Uyghur (AUC = 0.832) than in Han (AUC = 0.674), highlighting population-specific differences in genetic contribution to the ACS subtype classification.

To explore whether factors such as arterial narrowing and genetic sites influence the classification of ACS subtypes (unstable angina vs. myocardial infarction), we employed different combinations of variables for prediction. Initially, we used all 78 variables for prediction, including 12 clinical indicators and 66 genetic markers. The 12 clinical indicators included demographic variables (age, gender, and weight), cardiac function measures (heart function classification, Killip classification, BNP, and EF), and coronary artery assessments (number of narrowed vessels, left main, left anterior descending, circumflex, and right coronary narrowing). Details of the 66 genetic loci are listed in Supplementary Table 1. The prediction results demonstrated an internal AUC of 0.843 (95% CI: 0.808–0.878) for the Han population. Notably, the Uyghur population exhibited a higher AUC of 0.897 (95% CI: 0.848–0.946) (Figure 5A). Subsequently, predictions based solely on the 12 clinical indicators showed moderate accuracy for the Han and Uyghur populations. The AUC for the Han population was 0.790 (95% CI: 0.749–0.831), while the Uyghur population showed an AUC of 0.740 (95% CI: 0.673–0.806) (Figure 5B). Finally, using only the 66 genetic markers, the model achieved lower accuracy in the Han population (AUC = 0.674; 95% CI: 0.626–0.722) but performed better in the Uyghur population (AUC = 0.832; 95% CI: 0.774–0.889) (Figure 5C). These results suggest that these 66 genetic markers may play a more prominent role in distinguishing unstable angina from myocardial infarction in the Uyghur population. Detailed prediction performance metrics are provided in Table 3.

**TABLE 3:** Model performances based on different eigenvector combinations.

	AUC (95% CI)	Sensitivity	Specificity	PPV	NPV	Accuracy
Han all variables	0.843 (0.808, 0.878)	62.84%	92.17%	75.16%	86.80%	84.13%
Uyghur all variables	0.897 (0.848, 0.946)	76.12%	93.20%	75.00%	93.57%	89.59%
Han clinical indicators	0.790 (0.749, 0.831)	63.93%	83.30%	59.09%	85.96%	77.99%
Uyghur clinical indicators	0.740 (0.673, 0.806)	47.76%	95.20%	72.73%	87.18%	85.17%
Han genetic markers	0.674 (0.626, 0.722)	40.98%	88.25%	56.82%	79.85%	75.30%
Uyghur genetic markers	0.832 (0.774, 0.889)	71.64%	78.00%	46.60%	91.12%	76.66%

AUC, area under the receiver operating characteristic curve; PPV, positive predictive value; NPV, negative predictive value; 95% CI, 95 percent confidence interval.

## Discussion

ACS presents a significant challenge in cardiovascular medicine due to its diverse clinical manifestations and the necessity for tailored therapeutic approaches (Han et al., 2020). Research into the genetic basis of ACS has grown in recent years, aiming to utilize genetic variations to optimize treatment strategies (Fayed et al., 2023; Kong et al., 2022). This study explored the genetic variation associated with ACS subtypes in two distinct Chinese populations: the Han and Uyghur populations. The investigation involved 985 participants, shedding light on differences in clinical characteristics and genetic polymorphisms among different ACS subtypes. We made three key findings. First, genotypes of rs1135822 of *CYP2D6* and rs11209716 of *PTGER3* significantly differed in the various subtypes of ACS among the Han Chinese population, while genotypes of rs2514036 of *ACY3*, rs3758581 of *CYP2C19*, rs2238032 of *CACNA1C*, rs2108622 of *CYP4F2*, rs1057910 of *CYP2C9*, and rs7294 of *VKORC1* significantly differed in the various subtypes of ACS among the Uyghur Chinese population. Second, we identified the correlation between artery narrowing and genetic polymorphisms. Third, predictive models utilizing clinical indicators and genetic markers demonstrated varying accuracies, with a greater contribution of genetic markers found in the Uyghur population than in the Han Chinese population. These findings contribute to the advancement of personalized medicine within diverse ethnic groups within the Chinese population.

Prior research has reported associations between genetic polymorphisms in *CYP2D6* and variable responses to beta-blockers in patients with ACS. *CYP2D6* encodes a key enzyme in the cytochrome P450 family responsible for metabolizing approximately 25% of clinically used drugs (Romskaug et al., 2020), including beta-blockers such as metoprolol and carvedilol (Duarte et al., 2024). Variability in CYP2D6 activity—due to common polymorphisms—can lead to altered drug plasma levels and response (Meloche et al., 2020). The SNP rs1135822, located in the 3′ untranslated region (3′ UTR) of *CYP2D6*, has been studied in relation to post-transcriptional regulation and mRNA stability (Farberov et al., 2023). Although the SNP’s direct functional impact remains under investigation, it may influence gene expression indirectly via microRNA binding or other regulatory mechanisms. Although rs1135822 is not classified among the major functional *CYP2D6* alleles (e.g., *CYP2D6* *3, *4, and *10), several population-based studies have reported associations between this SNP and variability in drug metabolism, with conflicting results across ethnic groups (Castano-Amores et al., 2023; Castano-Amores et al., 2021). Notably, allele frequencies reported in Han Chinese from the Chinese Millionome Database (CMDB) are comparable to those observed in our Han ACS cohort (Qi et al., 2021), supporting the relevance of our finding to disease subtypes rather than the population background. In our study, rs1135822 was significantly associated with the ACS subtype distribution in the Han Chinese population, suggesting a potential ethnic-specific regulatory role that may merit further investigation.

Additionally, rs11209716 in *PTGER3*, a gene encoding the prostaglandin E receptor 3, has previously been associated with variability in drug response. Specifically, patients with the CC genotype of rs11209716 may experience a reduced risk of ACE inhibitor-induced cough compared to those with the TT genotype (Farberov et al., 2023; Liu et al., 2024). Although the mechanistic basis remains unclear, this SNP may affect receptor signaling or expression, potentially modulating the vascular tone or inflammatory pathways relevant to ACS pathophysiology. In our study, rs11209716 also showed significant variation among ACS subtypes in the Han population, but not in the Uyghur group. This finding underscores the importance of considering the genetic background in studies of cardiovascular disease etiology.

Uyghur people of China represent a Eurasian population with a specific genetic profile, comprising approximately 60% European and 40% East Asian ancestries (Chen et al., 2017; Ning et al., 2023). This study found that rs2514036 of *ACY3*, rs3758581 of *CYP2C19*, rs2238032 of *CACNA1C*, rs2108622 of *CYP4F2*, rs1057910 of *CYP2C9*, and rs7294 of *VKORC1* were significantly associated with ACS subtypes in the Uyghur population, but not in the Han population. However, after applying FDR correction for multiple comparisons, none of the associations remained statistically significant. Therefore, the findings should be interpreted as exploratory and hypothesis-generating. Previous studies have reported some similar results to ours. Zhang et al. reported that several other polymorphisms of *VKORC1* differed between Han and Uyghur populations and could affect drug metabolism (Zhang et al., 2018). Shi et al. found that genotypes of *CYP2C19* varied among different ethnic groups (Shi et al., 2021). Although allele frequency data for healthy Uyghur individuals are limited, prior regional studies (Zhang, 2021; Qi et al., 2022) suggest that the variant frequencies we observed are within expected population ranges, indicating that the observed associations are unlikely due to outlier background distributions. These findings suggest a distinct genetic background in the Uyghur population with implications for disease risk stratification and individualized treatment.

Previous studies revealed that coronary stenosis was correlated with the subtypes of ACS (Zhang et al., 2021; Kim et al., 2021). Genetic variants with documented pharmacologic or disease associations may influence ACS susceptibility, and an association between *MMP-9* polymorphisms and coronary artery stenosis has previously been reported in the Uyghur population (Wang et al., 2011). This study identified several differential genetic biomarkers associated with coronary artery narrowing in Han and Uyghur patients with ACS. In the Han Chinese population, significant differences were observed in genotypes such as rs8192935 and rs8192950 in *CES1* associated with right coronary artery narrowing. The rs1799853 variant in *CYP2C9* was linked to narrowing in the left anterior descending artery, while rs2242480 in *CYP3A4* and rs4149015 and rs4149056 in *SLCO1B1* were significantly associated with right coronary artery narrowing. In addition, rs730012 in *LTC4S* were significantly associated with narrowing in the left anterior descending, circumflex, and right coronary arteries. In the Uyghur population, significant genotype associations were identified, including rs2032582 in *ABCB1* with left main and left anterior descending artery narrowing, rs1801253 in *ADRB1* with circumflex artery narrowing, rs1135822 in *CYP2D6* with left main artery narrowing, and rs6065 in *GP1BA* with circumflex artery narrowing. Additionally, rs730012 in *LTC4S* and rs4149601 in *NEDD4L* were associated with right coronary artery narrowing, while rs2306283 in *SLCO1B1* was associated with narrowing in the left main and right coronary arteries. These results suggest that ACS patients exhibit differential genetic profiles across ethnic groups, which may contribute to variations in clinical presentations and disease progression. The polymorphisms identified in specific genes among Han and Uyghur Chinese populations could inform individualized treatment strategies and risk assessment based on coronary artery narrowing.

Machine learning models play a pivotal role in biomedical applications, including disease prediction, drug discovery, and personalized medicine. Several models have been developed using clinical and genetic variables to predict the treatment outcomes and stratify risk in ACS patients (Hu et al., 2019; Ke et al., 2022; Song et al., 2023). This study utilized predictive models incorporating clinical indicators and genetic biomarkers to classify ACS subtypes. All models were derived and tested in the same cohort, so reported accuracies are likely optimistic. Notably, the predictive model for the Uyghur population showed higher internal accuracy in predictions when using genetic markers alone than that for the Han population. The result suggests that genetic factors may contribute more prominently to the ACS subtype classification in the Uyghur population. Further prospective studies may corroborate that ethnicity-specific genetic information can potentially refine risk classification. The higher predictive performance observed in the Uyghur population, particularly when using genetic variables alone, may be attributed to several interrelated factors. Genetically, the Uyghur population is characterized by an admixed ancestry, comprising approximately 60% European and 40% East Asian genetic components (Pan et al., 2022). This unique genetic structure may result in greater inter-individual variability in genetic loci relevant to ACS, potentially increasing the discriminative power of genotype-based models. Additionally, differences in allele frequencies, linkage disequilibrium patterns, and gene–environment interactions between Uyghur and Han populations may also contribute to differential predictive capacity (Qi et al., 2021). Demographically, variations in lifestyle, diet, and clinical care access may further modulate disease presentation and treatment response (Liu et al., 2024). Together, these factors suggest that ethnicity-specific models warrant further investigation for future precision-medicine applications in admixed populations such as the Uyghur group.

The predictive models developed in this study demonstrated moderate-to-strong discriminatory ability in distinguishing ACS subtypes, particularly within the Uyghur population. The combined model using both genetic markers and clinical indicators achieved an AUC of 0.897. Because this value is based on internal cross-validation, its generalizability remains uncertain. This internal AUC lies within the upper range of values reported in previous studies that used clinical or genetic data to classify cardiovascular conditions. For instance, Lu et al. predicted ACS and associated mortality using electronic health record data and reported an AUC of 0.72 for predicting ACS and 0.84 for mortality (Lu et al., 2021). Meng et al. achieved an AUC of 0.843 for early detection of ACS (Meng et al., 2025). In the Han population, although the AUC based solely on genetic markers was lower (0.674), combining them with clinical indicators improved the model’s accuracy. These findings suggest that incorporating genetic markers may, after external validation, improve future diagnostic research, particularly in populations with distinct genetic backgrounds like the Uyghur group. However, these models presented should be interpreted as exploratory and hypothesis-generating. Validation in independent, prospective cohorts is necessary before clinical application. If confirmed, such models could support early risk stratification and help identify individuals at higher risk of progressing from unstable angina to myocardial infarction. Such risk-stratification tools could allow for more timely intervention and individualized management strategies (Zwartkruis et al., 2020).

Despite the valuable insights provided, several limitations should be noted. First, this was a single-center study, necessitating multicenter validation to confirm the findings across diverse populations. Second, the relatively limited sample size—particularly in the Uyghur population—may affect the generalizability of the results. External validation in larger, independent cohorts is needed to assess the reproducibility and robustness of the identified associations. Third, population stratification was not controlled using methods such as principal component analysis (PCA), which may lead to confounding due to the underlying ancestry-related genetic structure. PCA was not applied in this study because the genotyping panel included only 66 SNPs. These SNPs were selected based on their relevance to drug–response pathways rather than genome-wide coverage. This limited SNP density reduces the resolution needed to accurately assess and correct for ancestry-related structure. Future studies utilizing genome-wide genotyping arrays will be better positioned to account for population structure. Fourth, this study did not include a healthy control group for comparison of allele frequencies. Therefore, it remains unclear whether the observed SNP distributions are specific to ACS subtypes or reflect baseline ethnic variations. Inclusion of matched controls in future studies would help contextualize these findings and differentiate disease-specific associations from background genetic differences.

In conclusion, this study highlights distinct genetic and clinical features of ACS in the Han and Uyghur Chinese populations. It identifies population- and subtype-specific genetic polymorphisms, emphasizing the need for personalized therapeutic approaches. The predictive models, particularly those applied in the Uyghur population, demonstrate the potential utility for integrating genetic markers with clinical indicators to refine ACS classification. These findings support the advancement of precision medicine approaches for optimizing cardiovascular care in ethnically diverse populations.

## Data Availability

The original contributions presented in the study are included in the article/Supplementary Material, further inquiries can be directed to the corresponding author.
